# Evaluation and comparison of bioinformatic tools for the enrichment analysis of metabolomics data

**DOI:** 10.1186/s12859-017-2006-0

**Published:** 2018-01-02

**Authors:** Anna Marco-Ramell, Magali Palau-Rodriguez, Ania Alay, Sara Tulipani, Mireia Urpi-Sarda, Alex Sanchez-Pla, Cristina Andres-Lacueva

**Affiliations:** 1 Biomarkers & Nutrimetabolomics Laboratory, Nutrition, Food Science and Gastronomy Department, Food Technology Reference Net (XaRTA), Nutrition and Food Safety Research Institute (INSA-UB), Faculty of Pharmacy and Food Sciences, Pharmacy and Food Science Faculty, University of Barcelona, Barcelona, Spain; 2 CIBER Fragilidad y Envejecimiento Saludable [CIBERfes], Instituto de Salud Carlos III [ISCIII], Madrid, Spain; 3 Genetics, Microbiology and Statistics Department, Biology Faculty, University of Barcelona, Barcelona, Spain; 4 Statistics and Bioinformatics Unit, Vall d’Hebron Institut de Recerca (VHIR), Barcelona, Spain

**Keywords:** Bioinformatic tools, Database, Enrichment, HumanCyc, KEGG, Metabolite, Metabolomics, Over-representation analysis, Pathway, Reactome

## Abstract

**Background:**

Bioinformatic tools for the enrichment of ‘omics’ datasets facilitate interpretation and understanding of data. To date few are suitable for metabolomics datasets. The main objective of this work is to give a critical overview, for the first time, of the performance of these tools. To that aim, datasets from metabolomic repositories were selected and enriched data were created. Both types of data were analysed with these tools and outputs were thoroughly examined.

**Results:**

An exploratory multivariate analysis of the most used tools for the enrichment of metabolite sets, based on a non-metric multidimensional scaling (NMDS) of Jaccard’s distances, was performed and mirrored their diversity. Codes (identifiers) of the metabolites of the datasets were searched in different metabolite databases (HMDB, KEGG, PubChem, ChEBI, BioCyc/HumanCyc, LipidMAPS, ChemSpider, METLIN and Recon2). The databases that presented more identifiers of the metabolites of the dataset were PubChem, followed by METLIN and ChEBI. However, these databases had duplicated entries and might present false positives. The performance of over-representation analysis (ORA) tools, including BioCyc/HumanCyc, ConsensusPathDB, IMPaLA, MBRole, MetaboAnalyst, Metabox, MetExplore, MPEA, PathVisio and Reactome and the mapping tool KEGGREST, was examined. Results were mostly consistent among tools and between real and enriched data despite the variability of the tools. Nevertheless, a few controversial results such as differences in the total number of metabolites were also found. Disease-based enrichment analyses were also assessed, but they were not found to be accurate probably due to the fact that metabolite disease sets are not up-to-date and the difficulty of predicting diseases from a list of metabolites.

**Conclusions:**

We have extensively reviewed the state-of-the-art of the available range of tools for metabolomic datasets, the completeness of metabolite databases, the performance of ORA methods and disease-based analyses. Despite the variability of the tools, they provided consistent results independent of their analytic approach. However, more work on the completeness of metabolite and pathway databases is required, which strongly affects the accuracy of enrichment analyses. Improvements will be translated into more accurate and global insights of the metabolome.

**Electronic supplementary material:**

The online version of this article (10.1186/s12859-017-2006-0) contains supplementary material, which is available to authorized users.

## Background

Enrichment techniques for ‘omics’ data are key tools for understanding complex biological systems. These tools reduce the complexity of the data, improve interpretation and understanding of biological systems, and help to generate hypotheses. Although the number of tools for ‘omics’ is rapidly growing, suitable tools for metabolomics are still scarce. Most of the available tools for metabolomics data have been previously developed for other ‘omics’ technologies. These tools have been described in detail elsewhere [1–6].

Enrichment tools denote any analytic technique that benefits from molecular pathway or network information to gain insight into a biological system [4]. The most widely used methodology for performing such analysis is termed functional enrichment or over-representation analysis (ORA) [7]. This analysis looks for keywords or descriptors of the set of molecules of interest (e.g. those over-expressed) with respect to a background reference set (e.g. the whole genome/transcriptome/proteome/metabolome or the set of molecules detected by the technology employed) [1]. Classical enrichment analyses employ Fisher’s exact test, but many other enrichment methods have derived from it, e.g. hypergeometric, Kolmogorov–Smirnov or Wilcoxon statistical tests [6, 7].

To the best of our knowledge studies evaluating the performance of enrichment tools for metabolite sets do not exist yet. The aim of the present work will be to dissect, for the first time, these techniques. First of all, we have carried out an exploratory multivariate analysis of the state-of-the-art of bioinformatic tools for metabolomics sets to visualize their diversity. Then, we have examined the completeness of metabolite databases, the performance of ORA methods and accuracy of disease-based analyses. For these purposes, we have used datasets from metabolomic repositories, whose results have been already published in peer-reviewed journals. In addition, we have enriched selected metabolic pathways and then compared the outputs of these tools when using real datasets or enriched data. Thus the present study provides a global insight of the current status of bioinformatic tools for the analysis and interpretation of metabolite sets from metabolomic studies.

## Methods

### Datasets

The list of metabolites used in this work refers to five datasets from metabolomics studies in humans, already published in peer-reviewed journals, whose raw data, study information and the list of identified metabolites are available in MetabolomeXchange [8], an online portal of metabolomics repositories including MetaboLights [9], Metabolomics Workbench [10] and Metabolomic Repository Bordeaux. A brief summary of the datasets is shown in Table 1. These datasets correspond to the following publications: 1) Lanza et al. [11]; 2) Fiehn et al. [12]; 3) Kaluarachchi et al. [13]; 4) Hart et al. [14]; and 5) Zhu et al. [15]. P- and adjusted *p*-values were obtained from the original papers [11–15] and only metabolites with an adjusted *p*-value < 0.05 were used for tools comparison. The list of metabolites is shown as (Additional file 1: Table S1).

**Table 1:** Main characteristics of the datasets used, extracted from the repository MetabolomeXchange

Dataset	Repository reference	Condition of study	Metabolomic platform	Significant metabolites in publication	Total metabolites analysed by authors	Reference
1	ST000091	Type 1 diabetes	LC(RP)-MS	8	44	[11]
2	ST000383	Type 2 diabetes and obesity	GC-MS	27	106	[12]
3	MTBLS364	Smokers	NMR, LC(HILIC-/RP)-MS	81	–	[13]
4	MTBLS424	Breast cancer	NMR	22	25	[14]
5	ST000284	Colorectal cancer	LC(RP)-MS	42	113	[15]

*Abbreviations:* *GC* gas chromatography, *HILIC* hydrophilic interaction liquid chromatography, *LC* liquid chromatography, *MS* mass spectrometry, *NMR* nuclear magnetic resonance, *RP* reverse phase

### Search of metabolite identifiers

Bioinformatic tools for enrichment analysis require the metabolite name or code (identifier) from a metabolite database. Although Kyoto Encyclopaedia of Genes and Genomes (KEGG Compound) identifiers [16] are the most commonly used in metabolomics [3, 17], some tools prefer other database identifiers such as PubChem [18], BioCyc/HumanCyc (hereinafter only referred as HumanCyc) [19] or Chemical Entities of Biological Interest (ChEBI) [20].

We analysed the current completeness of the following metabolite libraries: Human Metabolome Database (HMDB) [21], KEGG, PubChem, HumanCyc, ChEBI, ChemSpider [22], the metabolic reconstruction Recon2 [23], METLIN [24] and Lipid Metabolites and Pathways Strategy (LipidMAPS) [25].

The list of significant metabolites from [11–15] was used to assess the completeness of these nine databases. The identification of metabolites had been carried out by original authors in all the datasets, and in some cases KEGG and HMDB identifiers were already provided by authors. Since the HMDB website provides links to other metabolite databases, we started the search of the identifiers on this site and then we extended this search to the LipidMAPS website and MetExplore [26] (for Recon2 codes). All the identifiers were then double-checked in the corresponding metabolite databases. If more than one metabolite identifier was found (e.g. in PubChem or ChEBI databases), we took all those identifiers and checked in ConsensusPathDB [27] which ones were recognized by the tool (not shown). When the stereochemistry of the metabolite was not specified, the most common chemical configuration was assumed. The complete list of metabolite identifiers is shown in Additional file 1: Table S1.

### Generation of enriched data

Most of the bioinformatic tools for the enrichment of metabolomics datasets accept a list of identifiers as output, while a lower number require quantitative data, e.g. concentration, fold change or peak intensity. Therefore we decided to work with a list of metabolites (name or identifier) from real datasets and enriched data to compare these tools. Although this approach do not allow us to assess some of the available tools, i.e. 3omics or PAPi, the use of simulated o synthetic data would have allowed us to examine a lower number of tools.

For data enrichment, the dataset with the most metabolites was selected (colorectal cancer, ST000284). The list of significant metabolites of this dataset (*n* = 42, obtained from [15]) was analysed with MetaboAnalyst [28], using the option ‘pathway analysis’. MetaboAnalyst’s output was examined and the three KEGG pathways that presented the lowest false discovery rate (FDR), based on the Benjamini-Hochberg procedure [29], were chosen for pathway enrichment: 1) Alanine, aspartate and glutamate metabolism; 2) Aminoacyl-tRNA biosynthesis, and 3) Arginine and proline metabolism.

The R package KEGGREST (v.1.17.0) [30] was employed to build an adjacency matrix [31] which linked the metabolites of the dataset (*n* = 113) with their corresponding KEGG pathways. One was assigned if the metabolite was part of that particular pathway, or 0 if not. Then five metabolites of each pathway were randomly sampled. Enriched data are shown in Additional file 2: Table S2.

### Statistical analysis

#### Similarity analysis

The most commonly used tools for metabolomics data enrichment were chosen for similarity analysis. This selection was formed by 3omics [32], BioCyc/HumanCyc [19], ConsensusPathDB, IMPaLA [33], Ingenuity® Pathway Analysis (IPA®, QIAGEN, Redwood City, CA), KEGG [16], MassTRIX [34], MBRole [1], MetaboAnalyst, Metabox [35], MetaCore™ (Thomson Reuters Inc., Carlsbad, CA), MetaMapp [36], MetExplore, MetScape [37], MPEA [38], PaintOmics [39], PAPi [40], PathVisio [41], Reactome [42], Small Molecule Pathway Database (SMPDB) [43], WikiPathways [44] and XCMS [45].

The main features of these tools were summarized on a binary matrix (Yes/No responses) including whether they 1) perform ORA, integration with other ‘omics’ or other enrichment analyses; 2) visualization of pathways, networks or other types of visualization; 3) use KEGG, BioCyc, Reactome, Wikipathways, SMPDB or other pathway databases; 4) are databases, programmable, open-source or online tools (Additional file 3: Table S3).

The similarity analysis was performed with the R package vegan (v.2.4–4) [46]. First, the Yes/No responses were transformed to 1 and 0, respectively. Then Jaccard’s coefficients were calculated and a non-metric multidimensional scaling (NMDS) was performed. This method plots dissimilar objects far apart in the ordination space and similar objects close to one another preserving ordering relationships among them [47].

#### Over-representation analysis

The performance of the tools that perform ORA in metabolomics datasets was assessed with the list of significant metabolites of the colorectal cancer dataset (ST000284) (*n* = 42) [15] and of enriched data. The comparative analysis of ORA tools was performed on tools employing KEGG, Reactome and HumanCyc as pathway database. The selected tools were ConsensusPathDB, HumanCyc, IMPaLA, MBRole, MetaboAnalyst, Metabox, MetExplore, MPEA, PathVisio and Reactome and the pathway mapping tool KEGGREST. Table 2 summarizes the main features of these tools and type of identifiers used. Analyses were performed following the guidelines of each tool.

**Table 2:** Summary of the tools used to assess the performance of over-representation (ORA) methods and their main characteristics (July 2017). Tools and databases are sorted alphabetically

Tool name	Tool version	Database used	Database version	Test used in this work	Platform	Input code	Website
ConsensusPathDB	32	HumanCyc KEGG Reactome	19.1 (06/2015) 80.0 (10/2016) 59 (12/2016)	Fisher’s exact test Fisher’s exact test Fisher’s exact test	Online	HumanCyc KEGG Reactome	http://cpdb.molgen.mpg.de/
HumanCyc	21.0	HumanCyc	21.0 (12/2016)	Fisher’s exact test	Online	Name	https://humancyc.org/
IMPaLA	10	HumanCyc KEGG Reactome	NA NA NA	Fisher’s exact test Fisher’s exact test Fisher’s exact test	Online	HumanCyc KEGG Reactome	http://impala.molgen.mpg.de/
IPA®	NA	IPA® disease	NA	Fisher’s exact test, Z-score	Java-based software	KEGG	
KEGGREST	1.17.0	KEGG	NA	–	R	KEGG	https://bioconductor.org/packages/release/bioc/html/KEGGREST.html
MBRole	2.0	HMDB disease HumanCyc KEGG	3.5 (01/2013) 17.1 (06/2013) 54.1 (05/2010)	Hypergeometric test Hypergeometric test Hypergeometric test	Online	HumanCyc KEGG	http://csbg.cnb.csic.es/mbrole2/
MetaboAnalyst	3.0	SMPDB disease KEGG	NA NA	Fisher’s exact test, hypergeometric test Fisher’s exact test, hypergeometric test	Online	KEGG	http://www.metaboanalyst.ca/
Metabox	NA	KEGG	NA	Hypergeometric test	R	PubChem	https://github.com/kwanjeeraw/metabox
MetaCore™	NA	MeSH and OMIM disease	NA	–	Online	PubChem	https://portal.genego.com/
MetExplore	2.11.2	HumanCyc KEGG	18.0 (02/2014) 74.0 (04/2015)	Fisher’s exact test Fisher’s exact test	Online	HumanCyc KEGG	http://metexplore.toulouse.inra.fr/metexplore2/
MPEA	(2010)	KEGG	(2010)	Hypergeometric test	Online	KEGG	http://ekhidna.biocenter.helsinki.fi/poxo/mpea
PathVisio	3.2.4	Reactome	54 (10/2015)	Z-score	Java-based software	KEGG	https://www.pathvisio.org/
Reactome	61	Reactome	61 (06/2017)	Fisher’s exact test	Online	KEGG	http://reactome.org/

Abbreviations: *NA* not available

The output of these tools was examined for the following metabolic pathways: 1) KEGG: the three aforementioned pathways; 2) Reactome: Metabolism of amino acids and derivates pathway; and 3) HumanCyc: tRNA charging pathway. Ranking (position in the list of pathways sorted by significance), total number of metabolites/pathway, number of hits/pathway, p- and adjusted *p*-value (generally FDR, calculated by the tools) were recorded from each output.

### Disease-based enrichment analyses

Disease-based enrichment analyses were performed by using the list of significant metabolites of the five datasets on: 1) MetaboAnalyst (SMPDB disease pathway database) [48]; 2) MBRole (HMDB disease database); 3) IPA® (Ingenuity® disease database); and 4) MetaCore™ (MeSH and OMIM disease databases). Disease, ranking (position according to their *p*-value), total number of metabolites/disease, number of hits/disease, p- and adjusted *p*-values were recorded from each output.

## Results

### Evaluation of the state-of-the-art of bioinformatic tools

**Fig. 1:**
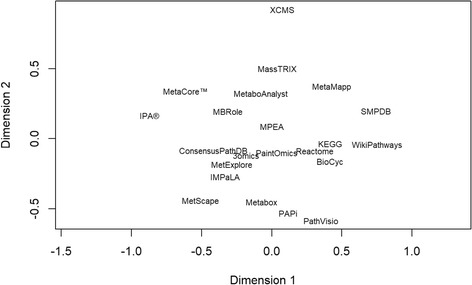
Non-metric multidimensional scaling (NMDS) plot of the most used tools for metabolomics data enrichment based on Jaccard’s distances. Additional file 3: Table S3 shows the main features of each tool

Figure 1 displays a similarity plot of the most commonly used bioinformatic tools. Tools were distributed all along the two dimensions revealing their diversity. The first dimension mainly separated tools that: 1) perform ORA and are non-open source, 2) perform ORA and are open-source, and 3) are a metabolite database. On the other hand, the second dimension mainly separated tools that: 1) perform metabolite identification, 2) perform ORA and are not programmable, and 3) perform ORA and are programmable. MetScape and MetaMapp, which only carry out data visualization, were distant in the plot.

### Evaluation of the completeness of metabolite databases

Metabolites of the five datasets were used to assess the completeness of the metabolite and pathway databases. Almost all the metabolites presented PubChem (97%), ChEBI (91%), METLIN (91%), KEGG (88%), ChemSpider (87%) and HMDB (86%) identifiers, and the 97% of the lipid subset had LipidMAPS identifiers. In some cases, KEGG, HumanCyc and Recon2 provided chemical class identifiers instead of a single identifier to certain metabolites, especially to the lipid subset (Additional file 1: Table S1 and Additional file 4: Table S4).

### Evaluation of over-representation methods

In general, ORA methods yielded consistent results using both real and enriched data in all the range of tools tested (Tables 3 and 4). Also similar results were obtained in paired analyses/tools such as MetaboAnalyst hypergeometric test - Fisher’s exact test, MBRole full - *Homo sapiens* database, MPEA top down - bottom up analyses, or ConsensusPathDB - IMPaLA tools, as expected (Tables 3 and 4).

**Table 3:** Evaluation of over-representation analysis (ORA) outputs of bioinformatic tools employing KEGG pathways. Real (from dataset ST000284) and enriched data were used. The number of total metabolites in the pathway, the number of hits, the ranking of the pathway among all the KEGG pathways (according to their significance), the *p*-value and the adjusted *p*-value were calculated by the tools

Tool	Data	Rank	Total metab.	Hits	*P*-value	Adjusted *p*-value
Alanine, aspartate and glutamate metabolism						
ConsensusPathDB	Real	2	28	8	3.77E-11	9.99E-10
	Enriched	2	28	7	3.76E-13	7.32E-12
IMPaLA	Real	2	28	8	3.77E-11	7.91E-09
	Enriched	2	28	7	3.76E-13	3.00E-10
KEGGREST	Real	NA	28	7	NA	NA
	Enriched	NA	28	7	NA	NA
MBRole (full database)	Real	2	24	8	3.47E-12	2.07E-10
	Enriched	1	24	7	7.23E-14	5.86E-12
MBRole (*Homo sapiens*)	Real	1	24	8	2.31E-11	1.50E-09
	Enriched	1	24	7	3.85E-13	2.00E-11
MetaboAnalyst (Fisher)	Real	1	24	7	3.91E-06	6.74E-05
	Enriched	1	24	7	6.21E-12	4.97E-10
MetaboAnalyst (hyper.)	Real	1	24	7	3.91E-06	6.74E-05
	Enriched	1	24	7	6.21E-12	4.97E-10
Metabox	Real	2	32	8	3.60E-11	5.22E-10
	Enriched	2	32	7	1.34E-13	1.27E-12
MetExplore	Real	3	NA	8	1.03E-08	4.32E-07
	Enriched	2	NA	7	4.42E-10	1.33E-08
MPEA (top down analysis)	Real	1	24	8	4.41E-11	0.660
	Enriched	1	24	7	5.01E-13	0.440
MPEA (bottom up analysis)	Real	1	24	8	1.01E-11	0.170
	Enriched	1	24	7	1.08E-12	1.00
Aminoacyl-tRNA biosynthesis						
ConsensusPathDB	Real	4	52	8	7.89E-10	1.05E-07
	Enriched	9	52	5	2.58E-07	1.12E-06
IMPaLA	Real	4	52	8	7.89E-09	8.74E-07
	Enriched	9	52	5	2.58E-07	1.13E-05
KEGGREST	Real	NA	52	8	NA	NA
	Enriched	NA	52	5	NA	NA
MBRole (full database)	Real	5	75	8	6.07E-08	1.20E-06
	Enriched	12	75	5	1.23E-06	8.30E-06
MBRole (*Homo sapiens*)	Real	5	75	8	3.75E-07	4.87E-06
	Enriched	6	75	5	3.95E-06	3.42E-05
MetaboAnalyst (Fisher)	Real	3	75	8	1.40E-05	3.75E-04
	Enriched	7	75	5	2.72E-05	3.11E-04
MetaboAnalyst (hyper.)	Real	3	75	8	1.40E-05	3.75E-04
	Enriched	7	75	5	2.72E-05	3.11E-04
Metabox	Real	4	56	8	4.28E-09	3.10E-08
	Enriched	4	56	5	8.69E-08	2.15E-07
MetExplore	Real	5	NA	8	1.55E-06	1.69E-06
	Enriched	7	NA	5	1.51E-05	4.52E-04
MPEA (top down analysis)	Real	3	53	8	5.32E-09	1.00
	Enriched	7	53	5	1.42E-06	1.00
MPEA (Bottom up analysis)	Real	5	53	8	7.12E-08	1.00
	Enriched	4	53	5	6.57E-08	1.00
Arginine and proline metabolism						
ConsensusPathDB	Real	9	76	7	2.79E-06	1.64E-05
	Enriched	4	76	6	3.94E-08	3.85E-07
IMPaLA	Real	9	76	7	2.79E-09	8.74E-07
	Enriched	4	76	6	3.94E-08	2.18E-06
KEGGREST	Real	NA	77	7	NA	NA
	Enriched	NA	77	6	NA	NA
MBRole (full database)	Real	3	82	10	2.59E-10	8.55E-09
	Enriched	2	82	8	9.30E-12	3.77E-10
MBRole (*Homo sapiens*)	Real	2	82	10	2.58E-09	8.38E-08
	Enriched	2	82	8	6.21E-11	1.61E-09
MetaboAnalyst (Fisher)	Real	2	77	9	6.69E-06	6.74E-05
	Enriched	2	77	8	8.61E-10	3.45E-08
MetaboAnalyst (hyper.)	Real	2	77	9	6.69E-06	6.74E-05
	Enriched	2	77	8	8.61E-10	3.45E-08
Metabox	Real	9	84	7	1.92E-05	6.18E-05
	Enriched	4	84	6	1.25E-08	5.96E-08
MetExplore	Real	2	NA	10	4.03E-08	1.69E-06
	Enriched	1	NA	8	3.34E-10	1.00E-08
MPEA (top down analysis)	Real	4	90	10	1.40E-08	1.00
	Enriched	2	90	7	1.09E-10	1.00
MPEA (bottom up analysis)	Real	2	90	10	2.24E-09	1.00
	Enriched	2	90	8	1.69E-10	1.00

NA means that information was not provided by the tool. Abbreviations: *Fisher* Fisher’s exact test, *hyper* hypergeometric test, *NA* not available

**Table 4:** Evaluation of over-representation analysis (ORA) outputs of bioinformatic tools employing Reactome and HumanCyc pathways. Real (from dataset ST000284) and enriched data were used. The number of total metabolites in the pathway, the number of hits, the ranking of the pathway among all the Reactome or HumanCyc pathways (according to their significance), the *p*-value and the adjusted *p*-value were calculated by the tools

Tool	Data	Rank	Total metab.	Hits	*P*-value	Adjusted *p*-value
Reactome						
Metabolism of amino acids and derivates						
ConsensusPathDB	Real	3	272	18	8.46E-14	4.55E-12
	Enriched	1	272	12	7.67E-15	5.75E-13
IMPaLA	Real	3	272	18	8.46E-14	4.21E-11
	Enriched	1	272	12	7.67E-15	1.02E-11
PathVisio	Real	NA	NA	NA	NA	NA
	Enriched	NA	NA	NA	NA	NA
Reactome	Real	9	283	18	1.03E-04	3.81E-03
	Enriched	1	283	12	8.18E-08	1.00E-05
HumanCyc						
tRNA charging						
ConsensusPathDB	Real	2	24	8	9.14E-12	1.92E-10
	Enriched	2	24	5	4.40E-09	1.30E-07
HumanCyc	Real	8	24	8	2.57E-05	0.002
	Enriched	18	24	5	7.90E-05	4.25E-03
IMPaLA	Real	2	24	8	9.14E-12	2.28E-09
	Enriched	2	24	5	4.40E-09	3.51E-07
MBRole (full database)	Real	4	64	8	8.38E-09	1.14E-06
	Enriched	47	64	4	3.58E-07	7.97E-06
MBRole (*Homo sapiens*)	Real	5	64	8	1.26E-04	2.44E-03
	Enriched	11	64	5	1.58E-04	1.52E-03
MetExplore	Real	1	NA	8	7.11E-07	7.75E-05
	Enriched	6	NA	5	4.45E-05	3.60E-04

NA means that information was not provided by the tool. Abbreviations: *NA* not available

Minor differences in the total number of metabolites/pathway and number hits/pathway were found. For instance, MPEA (Table 3) and MBRole (Table 4) presented a higher number of metabolites/pathway than the other tools. Other divergences were also observed, e.g. MPEA provided higher adjusted *p*-values values (nearly 1 in all the cases) than other tools, or not all the tools mapped the same metabolites of the dataset onto the queried pathways (not shown).

### Evaluation of disease-based libraries

The significant metabolites of the five datasets were used to analyse the accuracy of the SMPDB, HMDB, IPA®, MeSH and OMIM disease-based libraries. Outputs revealed that the diseases queried (diabetes type 1 and 2, obesity, respiratory alterations and breast and colorectal cancer) were not successfully identified by these tools, as they appeared in a low position in the list of potential diseases and most of the times they presented a *p* > 0.05 (Table 5).

**Table 5:** Disease-based enrichment analyses of the five datasets performed with MetaboAnalyst (SMPDB disease database), MBRole (HMDB disease database) and IPA® (in-house disease database) and MetaCore (based on MeSH and OMIM annotations). When the exact disease/condition of study was not obtained, a similar disease was selected

Dataset	Disease input	Disease output	Rank	Input number metabolites	Hits output	*P*-value	Adjusted *p*-value
MetaboAnalyst							
ST000091	Type 1 diabetes mellitus	Diabetes mellitus MODY	20	8	2	3.40E-02	5.84E-01
ST000383	Type 2 diabetes mellitus	Diabetes mellitus MODY	4	27	4	8.60E-03	6.69E-01
	Obesity	Obesity	31	27	1	9.07E-02	8.83E-01
MTBLS364	Smokers	–	–	81	–	–	–
MTBLS424	Breast cancer	Mammary tumour	30	22	2	4.08E-03	4.68E-02
ST000284	Colorectal cancer	Cervical/colon/ovarian cancer	46	42	1	8.47E-02	5.30E-01
MBRole							
ST000091	Type 1 diabetes mellitus	–	–	8	–	–	–
ST000383	Type 2 diabetes mellitus	Type 2 diabetes mellitus	8	27	3	1.16E-02	5.48E-02
	Obesity	Obesity	28	27	1	1.08E-01	1.48E-01
MTBLS364	Smokers	Lung Cancer	16	81	31	3.02E-02	9.25E-02
MTBLS424	Breast cancer	Lung Cancer	7	22	6	1.27E-04	1.09E-03
ST000284	Colorectal cancer	Colorectal cancer	44	42	1	5.19E-02	1.14E-01
IPA®							
ST000091	Type 1 diabetes mellitus	–	–	8	–	–	–
ST000383	Type 2 diabetes mellitus	Insulin resistance	21	27	3	6.10E-05	NA
	Obesity	Adipogenesis of fat	264	27	1	1.54E-02	NA
MTBLS364	Smokers	Cough	490	81	11	4.33E-02	NA
MTBLS424	Breast cancer	Gastric cancer	2	22	9	5.03E-11	NA
ST000284	Colorectal cancer	Colorectal cancer	3	42	11	2.31E-08	NA
MetaCore™							
ST000091	Type 1 diabetes mellitus	Type 1 diabetes mellitus	NA	8	0	NA	NA
ST000383	Type 2 diabetes mellitus	Type 2 diabetes mellitus	NA	27	7	NA	NA
	Obesity	Obesity	NA	27	1	NA	NA
MTBLS364	Smokers	Respiratory disorders	NA	81	1	NA	NA
MTBLS424	Breast cancer	Breast neoplasms	NA	22	0	NA	NA
ST000284	Colorectal cancer	Colorectal neoplasms	NA	42	13	NA	NA

Abbreviations: *NA* not available

## Discussion

Interpretation of metabolomic data is much less straightforward than that with genomic and proteomic datasets [36]. In the present work we have described the diversity of bioinformatic tools for metabolite sets and have evaluated their performance by exploring three features: the completeness of metabolite databases, ORA approaches and disease-based analyses. To that end, we have used five metabolite sets of blood biomarkers of different diseases obtained from LC-MS, GC-MS and NMR metabolomics approaches. This approach allowed minimizing the possible bias introduced by a given metabolomic platform and thus working with a wide range of metabolites.

Metabolomics is a developing field, thus bioinformatic tools designed to perform enrichment of metabolomics datasets are being developed and released by various groups using diverse statistical tests [3]. Our exploratory multivariate analysis mirrors the high diversity of the currently available tools for the analysis of metabolite sets.

To date about 30,000 endogenous metabolites have already been identified, but this number is rapidly increasing due to advances in high-throughput technologies [21]. Current metabolite databases do not have the full potential to quickly absorb these advances in the description of the endogenous metabolome yet, as not a single metabolite database used in this work covered the full list of significant metabolites of the five datasets. Among all the metabolites databases, PubChem was the one that covered more metabolites from the datasets. However, PubChem is a crowded compound database and presents duplicated metabolite entries, which might produce a larger number of false positives than searching against the KEGG database [49]. To address the low metabolite coverage of metabolite databases, some of them such as KEGG and HumanCyc assign chemical class identifiers to certain types of compounds, especially lipids such as phosphatidylcholines, sphingomyelins or triglycerides. For instance, KEGG coded phosphatidylcholines and sphingomyelins as ‘C00157’ and ‘C00550’, respectively, and HumanCyc as ‘PHOSPHATIDYLCHOLINE’ and ‘Sphyngomyelin (class)’.

Missing, ambiguous or redundant entries have been commonly found in public repositories [50]. Indeed metabolites with more than one PubChem, HMDB or ChEBI identifiers were found in this work, which reduce enrichment analyses’ accuracy. Several on-going initiatives on identifiers standardization such as BridgeDB and the Chemical Translation Service are trying to overcome redundancy [50–52]. Some tools such as MetaboAnalyst, ConsensusPathDB or PathVisio embrace these initiatives and accept different types of identifiers, which are then transformed into an internal identifier prior to the enrichment analysis [51]. However, this approach also presents different pitfalls. For instance, these tools usually transform the input code into KEGG identifiers, and thus certain types of metabolites such as lipids lose their uniqueness and become a chemical class KEGG identifier. Consequently bioinformatic tools analyse these lipids as a single entity, thereby losing the diversity of these metabolites.

KEGG and HumanCyc are the most used pathway libraries in metabolomics [3, 17] and Reactome is widely used in other ‘omics’ studies [53]. Thus we have evaluated and compared outputs of ORA methods that employ these pathway libraries. Some limitations prior to ORA analysis were found. For instance, despite the fact that almost all the metabolites of ST000284 dataset had a KEGG code, not all of them were mapped in a KEGG pathway. However, these compounds (e.g. 5-hydroxytryptophan and salicylurate) were mapped in other pathway databases such as Reactome, Wikipathways and SMPDB (not shown). In addition, the KEGG code for glutamic acid (C00025) was not recognized by MetaboAnalyst and the alternative suggested by the tool corresponded to the compound amphetamine (C07514).

The number of total metabolites and hits per pathway varied according to the tool used and those tools that employ the newer database versions (Table 2) presented the higher number of metabolites, as expected. Surprisingly, KEGGREST, a R package that provides an updated client interface to the KEGGREST server, did not provide the highest number of total metabolites among the tested KEGG pathways. Despite regular updates to some pathway databases, such as KEGG [16] or Reactome [42], being carried out, most of the tools evaluated do not use up-to-date database versions (Table 2) [54]. Wadi et al. performed an elegant review on the impact of outdated annotations on pathway enrichment analysis, which revealed that many software tools use functional information not updated for years, thereby strongly affecting the quality of the analyses [54].

We can conclude that current ORA methods, despite their differences, provide consistent, robust and reproducible results regardless of their analytic approach (statistical test, *p*-value adjustment or pathway database used), despite the limitations and small differences found between outputs. The most discordant result was obtained with MPEA, probably due to the fact that it employs a different method to handle many-to-many relationships that may occur between the query compounds and metabolite annotations [38].

Although we cannot recommend one tool over the others, we suggest choosing those tools that employ updated metabolite/pathway databases in order to obtain more complete results. Nevertheless, we also consider that the enrichment analysis must not be restricted to a single database or tool. The combined use of libraries such as KEGG, Reactome, HumanCyc or WikiPathways will increase the metabolome coverage and the statistical power of the enrichment analysis.

Disease-based enrichment analysis did not yield accurate results. Although we only used serum/plasma biomarkers, results with other types of biological samples would have been similar. On one hand, metabolite disease sets are not up-to-date. For instance, MetaboAnalyst and MBRole (SMPDB and HMDB disease databases, respectively) base their searches of literature dated between 1975 and 2008, as stated in the outputs of these tools. Since 2008, advances in high-throughput techniques have remarkably improved metabolomics analyses and, consequently, more knowledge about these diseases is available. As previously discussed, the use of not updated annotation sets strongly affect the quality of the analyses [54]. On the other hand, metabolites can overlap between unrelated physiopathological events since similar metabolic processes are altered [55]. This fact could complicate the development and accuracy of background sets for disease-based enrichment analysis.

Although extensive work in developing bioinformatic tools for metabolite sets has been carried out in recent years, more effort in improving metabolite/pathway databases and tools is still needed. On one hand, metabolite databases have to rapidly absorb new information from unstoppable advances in high-throughput technologies. On the other hand, enrichment methods should include a wider range of metabolite identifiers (e.g. LipidMAPS, ChemSpider or METLIN) and metabolite pathway databases in order to increase the metabolome coverage. For instance, the LipidMAPS Structure Database contains about 30,000 human endogenous lipids and 12,000 plant lipids, but also databases based on lipid metabolism and signalling pathways, MS/MS spectra and protein-related data [25, 56]. ChemSpider is a general chemical database and offers access to information for almost 25 million experimentally determined structures of natural and synthetic compounds [22]. However, similarly to PubChem, ChemSpider may lead to a high number of false positives [57]. The METLIN database includes nearly 1,000,000 molecules, ranging from lipids, steroids, plant & bacteria metabolites, small peptides, carbohydrates, exogenous drugs/metabolites and central carbon metabolites, and more than 200,000 MS/MS spectra [24]. Including these information sources in current bioinformatic tools would also involve more effort in the improvement of metabolite identifiers converters. Therefore, there is still a long way ahead to achieve complete metabolite and pathway databases and thus accurate enrichment analyses of metabolite sets.

## Conclusions

We have extensively reviewed, for the first time, the state-of-the-art of bioinformatic tools for the enrichment of metabolite sets from metabolomics studies, visualized their diversity, and examined their performance. The redundancy of identifiers, the use of chemical class identifiers and the incompleteness of metabolite databases and disease metabolite sets limit the extent of the analyses and reduce their accuracy. In general, ORA tools provided consistent results among tools revealing that these analyses are robust and reproducible regardless of their analytic approach. However, more work in the completeness of metabolite/pathway databases is required to get more accurate and global insights of the metabolome.

## Additional files

Additional file 1: Table S1. Full list of significant metabolites of the five datasets used in the present study (adjusted *P*-value < 0.05). Dataset A refers to dataset ST000091, B to ST000383, C to MTBLS364, D to MTBLS424 and E to ST000284. (XLSX 28 kb)

Additional file 2: Table S2. Enriched data and their main metabolite identifiers for ORA analysis. (XLSX 9 kb)

Additional file 3: Table S3. List of features of the tools analysed by multiple correspondence analysis. Abbreviations: N, no; Y, yes. (XLSX 12 kb)

Additional file 4: Table S4. Number of metabolites with identifiers of the following metabolite databases. Metabolite databases are sorted by the number of identifiers found. *LipidMAPS identifiers were only searched in lipids (*n* = 67), while the rest of identifiers were considered in all the metabolites of the datasets (*n* = 147). (DOCX 16 kb)

## Abbreviations

ChEBI: Chemical entities of biological interest
FDR: False discovery rate
HMDB: Human metabolome database
KEGG: Kyoto Encyclopaedia of genes and genomes
LipidMAPS: Lipid metabolites and pathways strategy
NMDS: Non-metric multidimensional scaling
ORA: Over-representation analysis
SMPDB: Small molecule pathway database
