# Supplementary material for: Evaluation and comparison of bioinformatic tools for the enrichment analysis of metabolomics data
Source: BMC Bioinformatics. 2018 Jan 2;19:1. doi: 10.1186/s12859-017-2006-0 (PMC5749025; doi:10.1186/s12859-017-2006-0)
Supplement: Supplementary file 4 — Number of metabolites with identifiers of the following metabolite databases. Metabolite databases are sorted by the number of identifiers found. *LipidMAPS identifiers were only searched in lipids (n = 67), while the rest of identifiers were considered in all the metabolites of the datasets (n = 147). (DOCX 16 kb) [file 12859_2017_2006_MOESM4_ESM.docx]

**Table S4.** Number of metabolites with identifiers of the following metabolite databases. Metabolite databases are sorted by the number of identifiers found. *LipidMAPS identifiers were only searched in lipids (n=67), while the rest of identifiers were considered in all the metabolites of the datasets (n=147).

|  | **Total** | **PubChem** | **METLIN** | **ChEBI** | **KEGG** | **ChemSpider** | **HumanCyc** | **HMDB** | **Recon2** | **LipidMAPS*** |
| --- | --- | --- | --- | --- | --- | --- | --- | --- | --- | --- |
| Metabolites with identifiers |  |  |  |  |  |  |  |  |  | - |
| Amino acids and related compounds | 35 | 34 | 33 | 34 | 33 | 34 | 32 | 34 | 29 | - |
| Organic acids | 19 | 18 | 18 | 19 | 19 | 18 | 17 | 18 | 13 | - |
| Carbohydrates | 7 | 7 | 7 | 7 | 7 | 7 | 4 | 6 | 3 | - |
| Lipids | 67 | 67 | 60 | 56 | 57 | 52 | 60 | 53 | 47 | 65 |
| Other compounds | 19 | 18 | 16 | 18 | 14 | 17 | 15 | 16 | 12 | - |
| Total | **147**  **(100%)** | **144**  **(98%)** | **134**  **(91%)** | **134**  **(91%)** | **130**  **(88%)** | **129**  **(87%)** | **128**  **(87%)** | **127**  **(86%)** | **104**  **(71%)** | **65**  **(97%)** |
| Unique identifiers |  |  |  |  |  |  |  |  |  |  |
| Amino acids and related compounds | 35 | 34 | 33 | 34 | 33 | 34 | 32 | 34 | 29 | - |
| Organic acids | 19 | 18 | 18 | 19 | 19 | 18 | 17 | 18 | 13 | - |
| Carbohydrates | 7 | 7 | 7 | 7 | 7 | 7 | 4 | 6 | 3 | - |
| Lipids | 67 | 67 | 60 | 42 | 20 | 52 | 21 | 53 | 20 | 65 |
| Other compounds | 19 | 18 | 16 | 18 | 14 | 17 | 15 | 16 | 12 | - |
| Total | **147**  **(100%)** | **144**  **(98%)** | **134**  **(91%)** | **120**  **(81%)** | **93**  **(63%)** | **129**  **(87%)** | **89**  **(61%)** | **127**  **(86%)** | **76**  **(52%)** | **65**  **(97%)** |
